# Rational design of deep eutectic solvents with low viscosities and multiple active sites for efficient recognition and selective capture of NH_3_

**DOI:** 10.1002/smo.20240045

**Published:** 2025-01-05

**Authors:** Lu Zheng, Saisai Ju, Siqi Fang, Hongwei Zhang, Zhenping Cai, Kuan Huang, Lilong Jiang

**Affiliations:** ^1^ National Engineering Research Center of Chemical Fertilizer Catalyst (NERC‐CFC) College of Chemical Engineering Fuzhou University Fuzhou Fujian China; ^2^ Qingyuan Innovation Laboratory Quanzhou Fujian China

**Keywords:** deep eutectic solvent, low viscosity, multiple active site, NH_3_ recognition, selective capture

## Abstract

Efficient recognition and selective capture of NH_3_ is not only beneficial for increasing the productivity of the synthetic NH_3_ industry but also for reducing air pollution. For this purpose, a group of deep eutectic solvents (DESs) consisting of glycolic acid (GA) and phenol (PhOH) with low viscosities and multiple active sites was rationally designed in this work. Experimental results show that the GA + PhOH DESs display extremely fast NH_3_ absorption rates (within 51 s for equilibrium) and high NH_3_ solubility. At 313.2 K, the NH_3_ absorption capacities of GA + PhOH (1:1) reach 6.75 mol/kg (at 10.7 kPa) and 14.72 mol/kg (at 201.0 kPa). The NH_3_ solubility of GA + PhOH DESs at low pressures were minimally changed after more than 100 days of air exposure. In addition, the NH_3_ solubility of GA + PhOH DESs remain highly stable in 10 consecutive absorption‐desorption cycles. More importantly, NH_3_ can be selectively captured by GA + PhOH DESs from NH_3_/CO_2_/N_2_ and NH_3_/N_2_/H_2_ mixtures. ^1^H‐NMR, Fourier transform infrared and theoretical calculations were performed to reveal the intrinsic mechanism for the efficient recognition of NH_3_ by GA + PhOH DESs.

## Graphical abstract

A class of GA + PhOH DESs with multiple active sites and low viscosities were prepared. GA + PhOH DESs exhibited extremely fast NH_3_ absorption rates, high NH_3_ solubilities, good regeneration performance and efficient recognition of NH_3_ from CO_2_, N_2_ and H_2_ in mixed gases. The selectivity of NH_3_/CO_2_ is higher than that of the majority of ILs and DESs.

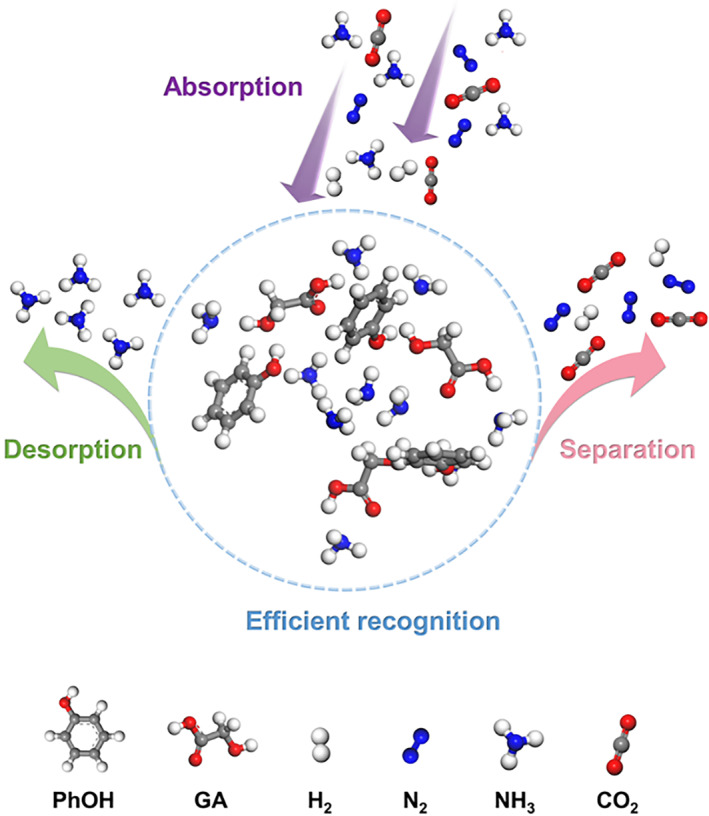

## 1 INTRODUCTION

The application of NH_3_ is so versatile that the employment of NH_3_ cannot be avoided. Apart from being employed for fertilizer production, NH_3_ can also be used for fabric dyeing and refrigerant production.[^1^, ^2^, ^3^] In addition, NH_3_ can be utilized as a fuel carrier due to its high H_2_‐storage density, and ease of transporting.^[4]^ Currently, the dominant approach of NH_3_ production is the Haber‐Bosch process, a conventional process for obtaining NH_3_ by reacting H_2_ with N_2_ at high temperature and pressure.^[5]^ Because of the reversibility limitation of the reaction, the conversion of H_2_ and N_2_ is incomplete, and The NH_3_ content in the product stream is limited to a maximum of 20 vol%. Worse still, approximately 3% by volume of the NH_3_ is difficult to separate using the commonly used physical condensation method because of the gas‐liquid equilibrium of NH_3_. Thus, the efficient recognition and selective capture of NH_3_ is conducive to improve the NH_3_ productivity. On the other hand, the extensive application of NH_3_ unavoidably results in the emission of NH_3_, which is an alkaline and odor‐intensive gas that may affect the atmosphere environment and human health.[^6^, ^7^] Thus, the efficient recognition and selective capture of NH_3_ is also conducive to reduce the air pollution.

Traditionally, water or inorganic acids are considered as the most economical absorbents for selective NH_3_ capture. Nevertheless, water's high heat capacity as well as volatility result in an energy‐intensive process for NH_3_ desorption. Inorganic acids may severely corrode the equipment, and the trapped NH_3_ is difficult to desorb as inorganic acids strongly interact with NH_3_. Therefore, there is an urgent need to design new absorbents that are safer and more effective for the recognition and capture of NH_3_. As a green absorbent of the latest generation, ionic liquids (ILs) are attracting increasing attention due to their unique characteristics, such as structural tunability, low volatility and outstanding solubility for various compounds.^[8]^ ILs have been broadly utilized for catalysis and separation,[^9^, ^10^, ^11^, ^12^] particularly, there are many brilliant studies on the capture of NH_3_ by ILs. For example, Yokozeki et al.^[13]^ pioneeringly investigated the NH_3_ solubility of several imidazole‐based ILs. Li's group^[14]^ further prepared functionalized imidazolium ILs by introducing −OH groups. The presence of −OH groups in ILs increases their viscosities, but the NH_3_ solubilities of ILs functionalized with −OH groups were significantly higher than those of ILs without −OH groups. Zeng's group^[15]^ prepared a group of Co‐containing imidazolium ILs and their NH_3_ absorption capacities were greatly improved compared to those of ILs without Co ions. Cai et al.^[16]^ fabricated a new type of IL by simply mixing Li salts with triethylene glycol (TEG). The −OH sites of TEG were activated thanks to the chelation of TEG with Li^+^, which also facilitates the trapping of NH_3_. Unfortunately, most ILs suffer from the disadvantages of complex and expensive preparation processes. The mass and heat transfer efficiencies of ILs in NH_3_ separation process are also interfered by their high viscosities, as continuous delivery of ILs in the towers and pipelines is necessary.

Deep eutectic solvents (DESs) not only have excellent physicochemical properties like ILs, but also have the merits of lower production expense and good biodegradability. Therefore, DESs are regarded as promising substitutes for ILs, and have been widely applied in the absorption and separation of gases such as CO_2_, H_2_S as well as SO_2_, for example,[^17^, ^18^, ^19^, ^20^] In recent years, notable advancements have also been made with regard to the utilization of DESs for NH_3_ separation.^[21]^ For example, Zhang and Huang^[22]^ prepared natural sugar—based DESs, and the resulting DESs exhibited high viscosities and high NH_3_ solubility as a result of the presence of the multi‐hydroxyl structures of natural sugars. Jiang's group^[23]^ designed a group of DESs using weak acids with different acidity as the hydrogen bond donors (HBD). The experimental results showed that the stronger the acidity of the HBD can facilitate the NH_3_ solubility of the DESs. Weak acidic groups interact with NH_3_ more strongly than the hydrogen bonding interaction. Zheng et al.^[24]^ designed non‐chlorinated DESs with multiple weak acidic sites, and the NH_3_ absorption capacity at low pressure was greatly improved. Zong et al.^[25]^ fabricated several Li‐based DESs, and the NH_3_ solubility of LiCl + ethylene glycol (1:3) DESs could reach 12.819 mol/kg at 303.15 K and 101.3 kPa. Cheng et al.^[26]^ constructed ternary metal‐based DESs, which dramatically improved the NH_3_ solubility at low pressure. The solubility of NH_3_ was 10.02 mol/kg at 313.2 K and 6.4 kPa. Recently, Sun et al.^[27]^ prepared ternary metal‐based DESs by introducing metal chlorides into resorcinol + ethylene glycol (1:2) DESs. The NH_3_ absorption capacity of MgCl_2_ + resorcinol + ethylene glycol (0.1:1:2) was improved by approximately 18.1%∼36.9% over pristine DES.

**FIGURE 1:**
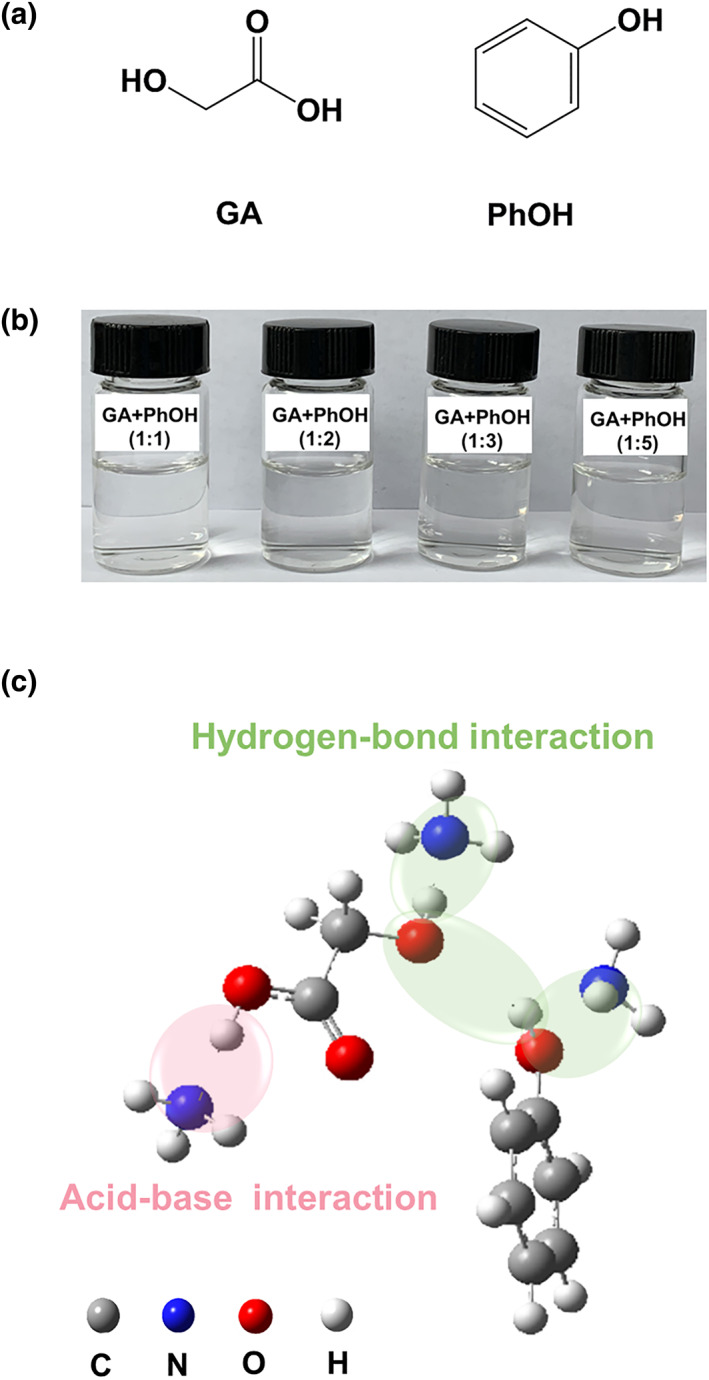
(a) Chemical structures of GA and PhOH; (b) photographs of GA + PhOH DESs; (c) schematic diagram of NH_3_ molecular recognition. GA, glycolic acid.

From the above analysis, it can be seen that the introduction of suitable active sites such as −OH groups, weak acidic sites and metal ions can effectively improve the ability of DESs for NH_3_ capture. Besides, the DESs with multiple active sites normally exhibit better NH_3_ capture ability than those with single active sites. Although there have been some DESs with multiple active sites reported for NH_3_ capture, most of them still have high viscosities. Here, we rationally designed a novel category of DESs that possess both multiple active sites and low viscosities for the capture of NH_3_. Specifically, the DESs are formulated by glycolic acid (GA) and phenol (PhOH) at different molar ratios, as shown in Figure 1. For GA, there is a −OH group and a −COOH group. Both groups have the potential for interaction with NH_3_; however, these interactions differ in their nature. The former interaction is of the hydrogen bonding kind, while the latter is characterized by a weak acid‐base interaction. For PhOH, there is aromatic −OH group that also can enables interact with NH_3_ through weak acid‐base interactions. Thus, the GA + PhOH DESs can offer multiple active sites for the recognition and capture of NH_3_. Interestingly, at 313.2 K, the GA + PhOH DESs show viscosities as low as 6.11 mPa·s, which is much lower than the viscosities reported for most DESs in the existing literature. Accordingly, a comprehensive investigation was conducted to examine the physical properties as well as NH₃ absorption behavior of GA + PhOH DESs. Additionally, the recognition mechanism of GA + PhOH DESs for NH_3_ was also examined by spectroscopy and theoretical calculations.

## 2 EXPERIMENTAL SECTION

### 2.1 Chemicals

Glycolic acid (GA, 99 wt.%) and phenol (PhOH, 99 wt.%) were offered by Shanghai Admas Reagent Co., Ltd. CO_2_, N_2_, H_2_ and NH_3_, all with a volume fraction of 99.99% were offered by Fujian Jiuce Gas Co., Ltd.

### 2.2 Preparation

A mixture of GA (0.05 mol, 3.84 g) and PhOH (0.05 mol, 4.80 g) was added to a flask, and stirred thoroughly at 333.2 K to obtain a uniform transparent liquid. The mixture was then subjected to vacuum drying at 333.2 K for one day to give the DES named as GA + PhOH (1:1), where 1:1 is the molar ratio of GA:PhOH. Similarly, mixtures with GA:PhOH molar ratios of 1:2, 1:3 and 1:5 were also prepared by the same procedures.

### 2.3 Characterizations

The moisture percentage of the samples were estimated on a Remco ZDY‐501 moisture analyzer, employing the Karl Fischer titration method. An Anton Paar 4100M digital densitometer was employed to assess the densities of samples, and the viscosities of samples were tested with the Anton Paar Lovis 2000 M/ME falling ball viscometer. A Netzsch DSC 214 Polyma analyzer was employed to determine the DSC curves within the temperature range of 233.2–353.2 K, with temperature variations occurring at a constant rate of 5 K/min. A Thermal Fischer Nicolet 6700 Hermo infrared spectrometer was applied to measure the Fourier transform infrared (FTIR) spectra, with a recording range of 4000 to 500 cm^−1^. The ^1^H nuclear magnetic resonance (^1^H‐NMR) spectra of the samples were detected on a Bruker Avance 600 spectrometer.

### 2.4 Gas solubility determinations

The gas solubility determination device used in this work was home‐made and its reliability has been supported by our previous work.[^28^, ^29^] Briefly, the device comprises two tanks, and their volumes were $V_{1}$ (m^3^) and $V_{2}$ (m^3^), and functioning as the storage and equilibrium tanks, respectively. The storage tank was used to hold the target gas internally and the equilibrium tank containing the liquid sample was used to perform the absorption of target gas. A steel pipe equipped with a high‐pressure valve connected two tanks. At the experimental temperature T (K), a specific quantity of target gas was filled into the storage tank and the pressure of the target gas was $P_{1}$ (kPa). Then the liquid sample with a mass of m (g) was added to the equilibrium tank, after which the internal air was evacuated. Next, the target gas was transferred to the equilibrium tank, and the pressure of the storage tank was reduced to $P_{2}$ (kPa). The target gas in the equilibrium tank was subjected to continuous absorption by the liquid sample. Once the pressure in the equilibrium tank stabilized for 10 min, the equilibrium absorption was achieved, and the pressure of the equilibrium tank was recorded as $P_{3}$ (kPa). The gas solubility w (mol/kg) was computed from Equation (1):

(1) $$w=\left[{\rho_{g}}^{P_{1},T}V_{1}-{\rho_{g}}^{P_{2},T}V_{1}-{\rho_{g}}^{P_{3},T}\left(V_{2}-w_{l}/{\rho_{l}}^{T}\right)\right]/m$$

where $\rho_{g}$ is the density of target gas at the temperature of T (K).

### 2.5 Gas mixture breakthrough experiments

Briefly, approximately 1 g of sample was added to a glass vessel. The gas mixture with a set composition was bubbled into the sample, accompanied by stirring of the liquid sample to promote gas diffusion. The gas flow rate was set to 60 ml/min. The real‐time components of the exit gas were detected by a gas chromatography‐mass spectrometry (GC‐MS).

### 2.6 Quantum chemistry calculations

A Gaussian 09 software was selected to carry out Quantum chemistry (QC) calculations.^[30]^ All the configurations were structurally optimized first and then frequency analyzed using the same method (B3LYP method in density functional theory [DFT]) and basis set (6–311 g++ (d,p)). Besides, the DFT‐D3 method was used for dispersion correction.^[31]^ The polarized continuum medium was with DMSO as the solvent was incorporated for structural optimization and frequency analysis.

### 2.7 Molecular dynamics simulation

An open‐source code CP2K/QUICKSTEP, applying the BLYP functionals of the triple‐ζ valence polarization basis set and the Goedecker‐TeterHutter pseudopotential basis set was utilized to implement the molecular dynamics (MD) simulations.^[32]^ A 14 × 14 × 14 Å^3^ box was first constructed, in which 5 GA, 5 PhOH, 15 NH_3_, 15 N_2_ and 15 H_2_ molecules were placed. Another 14 × 14 × 14 Å^3^ box was also constructed in which 5 GA, 5 PhOH, 15 NH_3_, 15 N_2_ and 15 CO_2_ molecules were placed for the simulations. The definition of the number of molecules is equal to the mass of the molecular clusters divided by the molecular weight of the molecular clusters. The mass of the molecular clusters is equal to the volume of the box multiplied by the density of the GA + PhOH DES. The MD simulations were conducted in an NVT ensemble using a Nosé‐Hoover chain thermostat with a finite temperature of 300 K and a time step of 1 fs. After the initial configuration was optimized for equilibrium, the radial distribution functions (RDFs) were calculated using simulation data. Note, that the initial 100 ps of data was ignored to ensure that the system had reached an equilibrium state.

## 3 RESULTS AND DISCUSSIONS

### 3.1 Physical properties

In this work, four mixtures GA + PhOH (1:1), GA + PhOH (1:2), GA + PhOH (1:3), and GA + PhOH (1:5) were prepared, and all of them are homogeneous and stable liquids at room temperature, as displayed in Figure 1. Firstly, the DSC curves of these mixtures were tested. As shown in Figure S1, the melting point of GA + PhOH (1:1) is not available within the temperature range of 243–353 K. The melting points of GA + PhOH (1:2), GA + PhOH (1:3), and GA + PhOH (1:5) are determined as 267 K, 281 and 280 K, respectively. Both GA and PhOH are solids at room temperature, with the melting points of 348 and 313 K,[^33^, ^34^] respectively. Therefore, GA + PhOH (1:1), GA + PhOH (1:2), GA + PhOH (1:3) and GA + PhOH (1:5) can be termed as DESs. Next, the water percentage of GA + PhOH DESs was tested. The moisture contents of GA + PhOH DESs ranged from 0.30 to 0.76 wt.%, as shown in Table S1. This indicates that the moisture effect on the physicochemical properties of GA + PhOH DES is negligible because of the extremely low water content.

**FIGURE 2:**
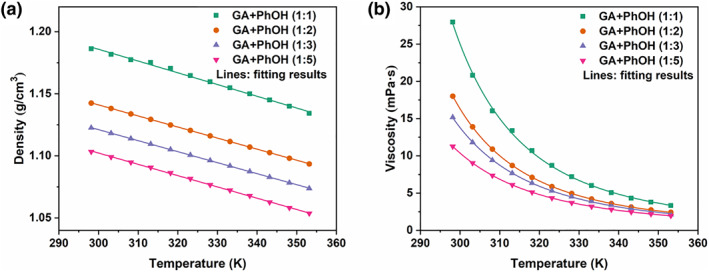
(a) Densities and (b) viscosities of GA + PhOH DESs. GA, glycolic acid; DESs, deep eutectic solvents.

Densities and viscosities are the basic properties of absorbents, especially the viscosities are related to the mass transfer efficiency of absorbents. The densities and viscosities of freshly prepared GA + PhOH DESs over the temperature range of 298.2∼353.2 K are provided in Figure 2a,b (see Tables S2 and S3 for data). A linear decrease in the densities of GA + PhOH DES can be observed with increasing temperature, which is compatible with the general trend for liquid absorbents. In addition, the densities decrease with increasing PhOH contents, which may be attributed to the lower density of PhOH in comparison to GA.[^35^, ^36^] The viscosities of GA + PhOH DESs also decrease with increasing temperature. However, the viscosities show nonlinear correlation with temperature changes. The decrease in viscosities is observed at a fixed temperature if the PhOH contents increase, which may be a result of the fact that fewer intermolecular hydrogen bonds are formed at higher PhOH contents. Notably, at 298.2 K, the viscosities of GA + PhOH DESs are only 11.27∼27.95 mPa·s. These values are much lower than those reported for the majority of liquid absorbents in the existing literature, and should be very favorable for mass transfer during NH_3_ capture. The density as well as viscosity data can be fitted by Equations (2) and (3), respectively, and Table S4 illustrates the relevant fitted parameters.

(2) ρ=a+bT

(3) $$\eta =\eta_{0}\;\exp\frac{D}{T-T_{0}}$$

where ρ (g/cm^3^) represents the density of DES; η (mPa · s) represents the viscosity of DES; T (K) represents experimental temperature. a, b, $T_{0}$, $\eta_{0}$ as well as D represent empirical parameters to be fitted.

### 3.2 NH_3_ capture performance

**FIGURE 3:**
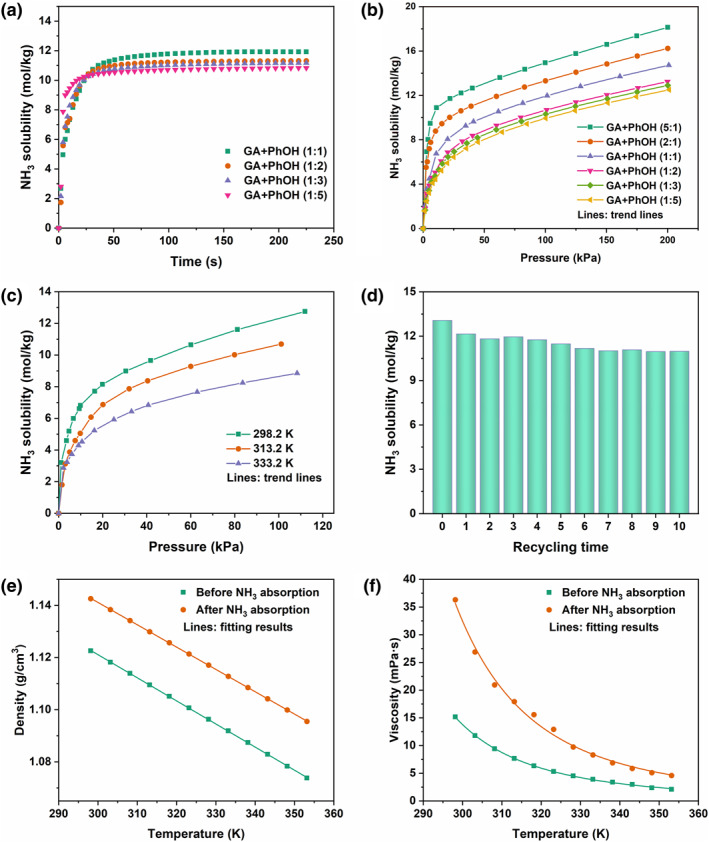
(a) NH_3_ absorption rate and (b) NH_3_ solubility in GA + PhOH DESs at 313.2 K; (c) NH_3_ solubility in GA + PhOH (1:2) at different temperatures; (d) recycling of NH_3_ absorption and desorption by GA + PhOH (1:2) (absorption condition: 298.2 K and about 101 kPa; desorption condition: 333.2 K and about 0.01 kPa for 2 h); (e) densities and (f) viscosities of GA + PhOH (1:3) before and after NH_3_ capture. GA, glycolic acid; DESs, deep eutectic solvents.

Low viscosities of absorbents are normally beneficial for the mass transfer in industrial applications, so the rates of NH_3_ absorption in GA + PhOH DESs were tested firstly. The amount of NH_3_ absorbed was calculated as a function of time. As depicted in Figure 3a, the rates of NH_3_ absorption in all the four DESs are notably rapid, almost reaching equilibrium within 27∼51 s. The equilibrium rates are negatively correlated with the contents of PhOH in DES, that is, the higher the contents of PhOH in DESs, the faster NH_3_ absorption of DESs. In other words, GA + PhOH DESs with lower viscosities show faster absorption rates of NH_3_.

Next, the NH_3_ solubility profiles of GA + PhOH DESs were measured at 313.2 K. The results are illustrated in Figure 3b, and the data are presented in Table S5. The NH_3_ solubility gradually increase with the increase in pressures, and there is a nonlinear relationship between the solubility of NH_3_ and pressures. Such profiles suggest that there is a chemical interaction between GA + PhOH DESs and NH_3_. Furthermore, with the increase of GA contents in GA + PhOH DESs, the NH_3_ absorption capacities also increase. For example, at around 200 kPa, the NH_3_ absorption capacities of GA + PhOH (1:1), GA + PhOH (1:2), GA + PhOH (1:3) and GA + PhOH (1:5) are 14.72, 13.24, 12.90 and 12.50 mol/kg, respectively. This indicates that NH_3_ interacts more strongly with GA compared to with PhOH, thus GA is a key component in the recognition and capture of NH_3_ by GA + PhOH DESs. Further, GA + PhOH (5:1) and GA + PhOH (2:1) with GA:PhOH molar ratios greater than 1 were prepared in order to enhance the NH_3_ absorption capacities of GA + PhOH DESs and their NH_3_ absorption capacities were determined. As depicted in Figure 3b, at 200 kPa, the NH_3_ absorption capacities of GA + PhOH (5:1) and GA + PhOH (2:1) are 18.12 mol/kg and 16.24 mol/kg, respectively, which are much higher than those of the GA + PhOH DESs with GA:PhOH molar ratios lower than 1. In addition, the solubility of NH_3_ in DESs are very considerable no matter at low or high pressures. For example, at 313.2 K, the NH_3_ absorption capacities of GA + PhOH (1:1) are 6.75 mol/kg (at 10.7 kPa) and 14.72 mol/kg (at 201.0 kPa), respectively. These values significantly exceed those reported for a majority of liquid absorbents and solid adsorbents. The capacities of NH_3_ absorption by various liquid absorbents and solid adsorbents are summarized in Table 1 [^14^, ^26^, ^37^, ^38^, ^39^, ^40^, ^41^, ^42^, ^43^, ^44^, ^45^, ^46^, ^47^, ^48^, ^49^, ^50^, ^51^, ^52^, ^53^, ^54^, ^55^, ^56^, ^57^] and Table S6,[^58^, ^59^, ^60^, ^61^, ^62^, ^63^, ^64^, ^65^, ^66^, ^67^, ^68^, ^69^], respectively. In particular, the NH_3_ solubility of PhOH‐containing liquid absorbents such as EtOHACl + PhOH (1:3), [TMPDA]Cl_2_ + PhOH (1:7), and Res/PhOH (1:3) in Table 1 are lower than that of the GA + PhOH DESs. EtOHACl, [TMPDA]Cl_2_ and Res have two chemical active sites, respectively. However, GA has multiple active sites, However, GA has multiple active sites, which may be beneficial in improving the solubility of NH_3_ by GA + PhOh DES compared to EtOHACl + PhOH DES, [TMPDA]Cl_2_ + PhOH DES and Res/PhOH DES. The intrinsic mechanism of NH_3_ capture by GA will be revealed in the NH_3_ recognition mechanism section.

**TABLE 1:** Comparison of NH_3_ absorption capacities for different ILs and DESs.

Absorbent	*T* (K)	*P* (kPa)	NH_3_ capacity (mol/kg)	Ref.
GA + PhOH (1:2)	298.2	9.9	6.82	This work
		111.9	12.75	
GA + PhOH (1:1)	313.2	10.7	6.75	This work
		101.4	11.97	
[APH]NO_3_‐Res (2:1)	293.0	100.0	14.88	[37]
EtOHACl + PhOH (1:3)	298.2	11.2	5.50	[38]
		100.4	10.70	
EaCl + CoCl_2_ + Gly (1:0.8:3)	298.2	6.8	10.24	[26]
		103.0	17.55	
ImCl + Gly (1:2)	298.2	10.0	4.45	[39]
		101.3	12.93	
[TMPDA]Cl_2_ + PhOH (1:7)	298.2	13.3	4.49	[40]
		93.1	9.18	
EaCl‐Gly(1:2)‐6% OHP[5]	298.2	10.0	2.39	[41]
		100.0	10.84	
LiCl/EG (1:3)	303.2	101.3	12.82	[42]
GI‐AT (1:2)	303.2	100.0	5.30	[43]
MgCl_2_/Res/EG (0.25:1:2)	303.2	101.3	12.12	[44]
[Bmim]_2_[CuCl_4_]	303.2	100	10.10	[45]
MAA + tetrazole (2:1)	313.2	10.0	4.64	[46]
		101.3	7.94	
[4‐MeOHPy][NTf_2_]	313.2	21.1	6.01	[47]
		99.9	8.79	
NH_4_SCN + Gly (2:3)	313.2	7.4	1.89	[48]
		101.3	10.36	
[Me_2_COH_2_N]Cl:U (1:1)	313.2	100.0	2.08	[49]
ChCl/Res/Gly (1:3:5)	313.2	101.3	7.65	[50]
[EtOHmim][BF_4_]	313.2	115.6	3.07	[14]
2‐NH_2_Py/Res (1:2)	313.2	101.3	9.59	[51]
TetrZ + 1,2,3‐ tri(1:4)	313.2	101.3	14.06	[52]
Res/PhOH (1:3)	313.2	101.3	10.60	[53]
[2‐mPy][NTf_2_]	313.2	100.0	8.24	[54]
EaCl/Res/Gly (1:4:5)	313.2	101.3	10.65	[55]
[EtOHim][NTf_2_]	313.0	100.0	7.94	[56]
[1, 2, 3‐TrizH_2_][CF_3_SO_3_]_2_	313.0	100.0	15.41	[57]

Abbreviations: DESs, deep eutectic solvents; ILs, ionic liquids.

Subsequently, the effect of temperature on the absorption of NH_3_ by GA + PhOH DESs was explored as represented by GA + PhOH (1:2). The results are depicted in Figure 3c and the data are shown in Table S7. The NH_3_ solubility of GA + PhOH (1:2) are negatively correlated with the temperature changes at a fixed pressure, which suggests that NH_3_ absorption is exothermic. Therefore, a decrease in temperatures favors NH_3_ absorption, while an increase in temperatures favors NH_3_ desorption. Indeed, long‐lasting regeneration performance is desired for ideal NH_3_ absorbents. Therefore, consecutive absorption‐desorption experiments were performed for GA + PhOH DES. Given that high temperatures are conducive to NH_3_ desorption, the stability of GA + PhOH (1:2) at 333.2 K was first tested. As shown in Figure S2, the mass loss rate of GA + PhOH (1:2) is about 2.6% after a continuous N_2_ purge at 333.2 K for 8 h, which is almost negligible, indicating that 333.2 K is an acceptable desorption temperature. The NH_3_ absorption capacities of GA + PhOH (1:2) during 10 consecutive absorption‐desorption cycles are presented in Figure 3d. It is found that after 10 cycles, the NH_3_ solubility of GA + PhOH (1:2) was found to be 10.98 mol/kg at 298.2 K and 101 kPa, which is 83.3% of the initial NH_3_ absorption capacity, reflecting the good regeneration performance of GA + PhOH DESs. The lost NH_3_ capacity may be due to the fact that 333.2 K is a relatively mild desorption temperature at which the absorbed NH_3_ is difficult to be fully released.

Notably, the densities and viscosities of GA + PhOH (1:3) after NH_3_ absorption at 3 kPa were tested. As presented in Figure 3e,f (see Tables S2 and S3 for data), the GA + PhOH (1:3) after NH_3_ absorptoin show only slight increase in densities and viscosities compared to fresh GA + PhOH (1:3), which is favorable in industrial applications.

### 3.3 Long‐term stability test towards air

**FIGURE 4:**
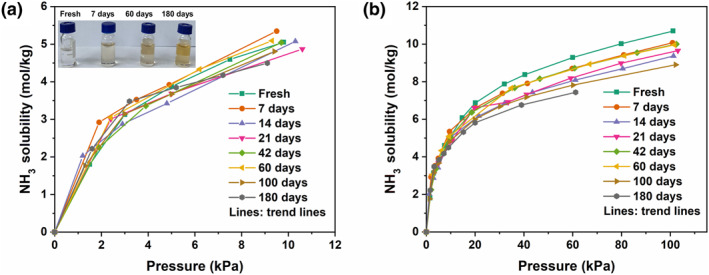
NH_3_ solubility of GA + PhOH (1:2) exposed to air at 313.2 K within (a) 10 kPa and (b) 100 kPa (inset: images of air exposure at different times). GA, glycolic acid.

To determine the impact of air on the NH_3_ absorption behavior in GA + PhOH DESs, the NH_3_ solubility of the GA + PhOH (1:2) were measured after different exposure times in air at 313.2 K. As shown in Figure 4, it is easy to find out that the effect of air on NH_3_ solubility of GA + PhOH DESs in the low‐pressure range is insignificant. The differences in NH_3_ solubility induced by GA + PhOH DES after different exposure time in air are mainly concentrated in the relative high‐pressure range. Within 5 kPa, the NH_3_ solubility of GA + PhOH (1:2) after 180 days of exposure in air are almost unchanged. The NH_3_ absorption capacity of fresh GA + PhOH (1:2) is 10.70 mol/kg at 100 kPa, whereas the NH_3_ absorption capacity of GA + PhOH (1:2) after 60 days of exposure to air is 9.96 mol/kg, which is 93.11% of the pristine NH_3_ solubility. Despite a change in color from colorless to yellow with increased exposure time in the GA + PhOH (1:2), there is no evident impairment in its ability to recognize NH_3_, particularly under conditions of low pressure. This result suggests that GA + PhOH DESs exhibit excellent long‐term stability toward air, which contributes to the reduction of their cost of service.

### 3.4 NH_3_ selective recognition

**FIGURE 5:**
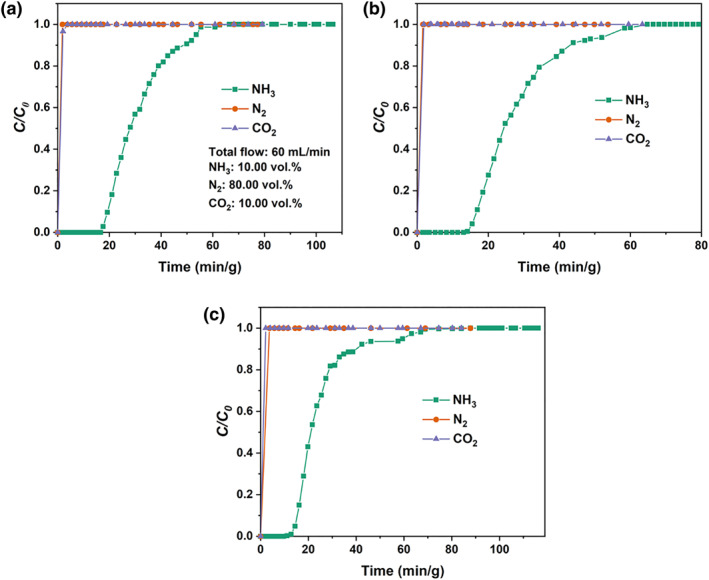
Breakthrough curves for NH_3_/N_2_/CO_2_ mixed gas in GA + PhOH (1:2) at (a) 298.2 K, (b) 313.2 K and (c) 333.2 K. GA, glycolic acid.

The selective recognition of NH_3_ absorbent is a major indicator of its overall performance. Therefore, we investigated the breakthrough curves of NH_3_/N_2_/CO_2_ mixtures in GA + PhOH (1:2) since separating NH_3_ from CO_2_ and N_2_ is concerned with the utilization of NH_3_. As demonstrated in Figure 5, the penetration of N_2_ and CO_2_ is very rapid and almost instantaneous. Whereas NH_3_ penetrates more slowly than N_2_ and CO_2_, the normalized time for NH_3_ to start penetrating in GA + PhOH (1:2) are 18, 14, and 11 min/g at 298.2, 313.2, and 333.2 K, respectively. Since NH_3_ absorption capacities decrease with increasing temperature, high temperature leads to fast breakthrough of NH_3_. Overall, this also demonstrates that GA + PhOH (1:2) has an extremely high NH_3_ selective recognition ability towards N_2_ and CO_2_, which is not limited by temperature changes. Furthermore, the NH_3_ penetration capacities of GA + PhOH (1:2) at 298.2, 313.2, and 333.2 K were calculated to be 7.48, 6.21, and 5.46 mol/kg, respectively. At 313.2 K, the NH_3_/N_2_/CO_2_ penetration curves of the DESs with different GA:PhOH molar ratios are also provided. As presented in Figure S3. The normalized time for the onset of NH_3_ penetration for GA + PhOH (1:1), GA + PhOH (1:2), GA + PhOH (1:3) and GA + PhOH (1:5) are 19, 14, 10, and 9 min/g, respectively. However, the instantaneous penetration of N_2_ and CO_2_ indicates that it is difficult for the GA + PhOH DESs to recognize N_2_ and CO_2_. Moreover, GA + PhOH DESs have high NH_3_ recognition towards N_2_ and CO_2_, which is not affected by changing the GA/PhOH molar ratio.

**FIGURE 6:**
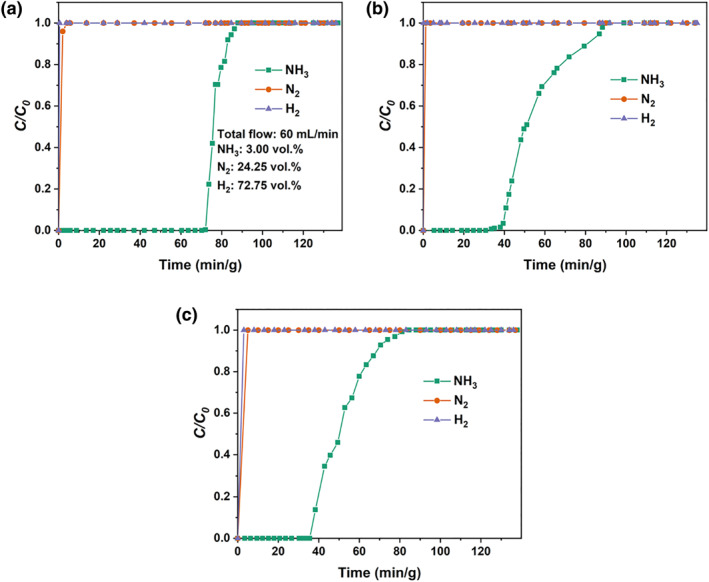
Breakthrough curves for NH_3_/N_2_/H_2_ mixed gas in GA + PhOH (1:2) at (a) 298.2 K, (b) 313.2 K and (c) 333.2 K. GA, glycolic acid.

In addition, the circulating gases in the synthetic NH_3_ industry are usually accompanied by H_2_ and N_2_; therefore, it is obligatory to examine the selective recognition of NH_3_ by GA + PhOH DES in NH_3_/N_2_/H_2_ mixtures. The contents of NH_3_, N_2_, and H_2_ were 3.00, 72.75, and 24.45 vol.%, respectively, to simulate the composition of the circulating gases in the synthetic NH_3_ process. As presented in Figure 6, it can be seen that NH_3_ penetrates very slowly, yet, the penetration time of N_2_ and H_2_ is almost zero, which reflects the high NH_3_ selective recognition of GA + PhOH (1:2) in the NH_3_/N_2_/H_2_ mixtures. Furthermore, the NH_3_ penetration capacities of GA + PhOH (1:2) at 298.2, 313.2 and 333.2 K were calculated to be 5.58, 3.54 and 3.36 mol/kg, respectively.

Although GA + PhOH DES is difficult to recognize for low concentrations of CO_2_, N_2_ and H_2_, gas capture experiments of GA + PhOH (1:2) for pure CO_2_, H_2_, and N_2_ were indeed performed. However, the N_2_ and H_2_ absorption capacities of GA + PhOH (1:2) were below the lower limit of detection of the instrument and could not be determined. The solubility of GA + PhOH (1:2) for pure CO_2_ at 313.2 K are shown in Figure S4 (see Table S8 for data). The pure CO_2_ absorption capacities of GA + PhOH (1:2) are extremely low, with a CO_2_ absorption capacity of 0.042 mol/kg at 100 kPa, which is only 0.4% of the NH_3_ absorption capacity. Furthermore, the linear correlation between CO_2_ absorption capacities and pressure changes indicates that there is no chemical interaction between GA + PhOH (1:2) and CO_2_, which also accounts for its extremely low CO_2_ absorption capacities. The NH_3_/CO_2_ selectivities of GA + PhOH (1:2) at 298.2 K, 313.2 K as well as 333.2 K were 228 (at 111.9 kPa), 252 (at 101.1 kPa), and 340 (at 108.3 kPa), respectively, which were higher than those of most liquid absorbents, see Table S9 for comparison.

### 3.5 NH_3_ recognition mechanism

**FIGURE 7:**
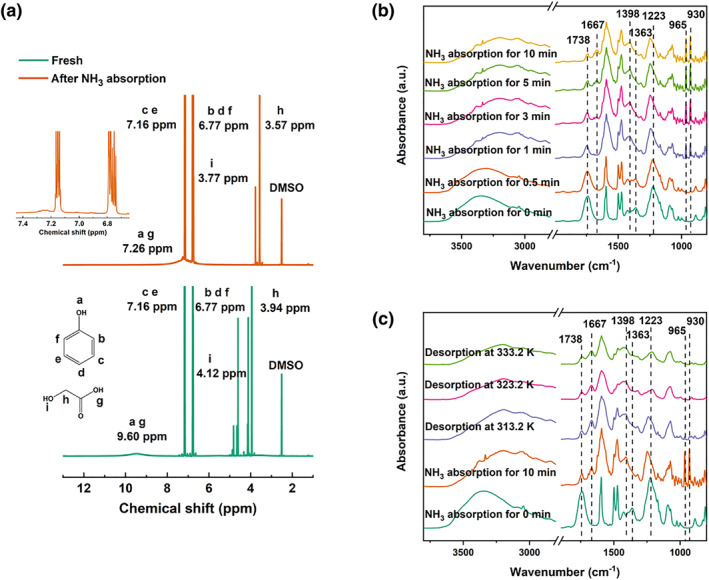
(a) ^1^H‐NMR spectra of GA + PhOH (1:2) before and after NH_3_ absorption; (b) in situ FTIR spectra of GA + PhOH (1:2) during NH_3_ absorption (313.2 K, 10 vol.% of NH_3_ balanced in N_2_); (c) in situ FTIR spectra of GA + PhOH (1:2) during NH_3_ desorption (20 ml/min of N_2_ purge). GA, glycolic acid; FTIR, Fourier transform infrared.

GA + PhOH DESs can efficiently and selectively recognize NH_3_. To explore the intrinsic mechanism of NH_3_ recognition, the ^1^H‐NMR spectra of GA + PhOH (1:2) after NH_3_ absorption were first determined. As illustrated in Figure 7a, after absorption of NH_3_, the proton peaks of −OH in PhOH and −COOH in GA shift from 9.60 to 7.26 ppm and the proton peak of −OH in GA shifts from 4.12 to 3.77 ppm. The changes in the ^1^H‐NMR spectra before and after absorption indicate that −OH in PhOH, −COOH and −OH in GA all interact with NH_3_. From the results of the in situ FTIR spectra shown in Figure 7b, similar conclusions can be drawn. For the pristine GA + PhOH (1:2), the peaks at 1738 and 1363 cm^−1^ as well as 1223 cm^−1^ characteristic for −COOH in GA, −OH in PhOH and C−OH in GA,[^70^, ^71^, ^72^] respectively. During NH_3_ absorption, there is a decline in the intensity of peak located at 1738 cm^−1^, the peak at 1363 cm^−1^ disappears, and the peak at 1223 cm^−1^ gradually shifts to 1248 cm^−1^. In addition, new peaks gradually appear at 1667 and 1398 cm^−1^, which are characteristic peaks of ${\text{NH}}_{4}^{+}$.[^73^, ^74^] Two other new peaks located at 965 cm^−1^ as well as 930 cm^−1^ are also observed, which are associated with the physical absorption peaks of NH_3_.^[75]^ These results indicate that −OH in PhOH, −COOH and −OH in GA, can all interact with NH_3_, which is conforming to the multisite active site concept. Furthermore, GA + PhOH (1:2) can chemically interact with NH_3_ because of the generation of ${\text{NH}}_{4}^{+}$, and this finding is supportive of the results of obtained in NH_3_ capture experiments. Similar conclusions can be obtained from the FTIR spectra as shown in Figure S5. Besides, the difference in the FTIR spectral curves between fresh GA + PhOH (1:2) and GA + PhOH (1:2) exposed to air for 14 days is hardly visible, which verifies the high stability of GA + PhOH DES towards air exposure. The spectral profiles of fresh GA + PhOH (1:2) and GA + PhOH (1:2) exposed to air for 14 days are similar after absorbing NH_3_. This also accounts for the long‐term stability of GA + PhOH DES towards air.

During NH_3_ desorption, the in situ FTIR curve is shown in Figure 7c. As the desorption temperature increases to 333.2 K, the peaks at 1667 cm^−1^ as well as 1398 cm^−1^ gradually diminish, and the peaks at 965 cm^−1^ as well as 930 cm^−1^ disappear. This demonstrates that the physically absorbed NH_3_ is easily released with increasing temperature. However, the absorbed NH_3_ could not be completely released at 333.2 K since GA + PhOH DESs can chemically interact with NH_3_, which is consistent with the result of the absorption‐desorption cycle experiments.

**FIGURE 8:**
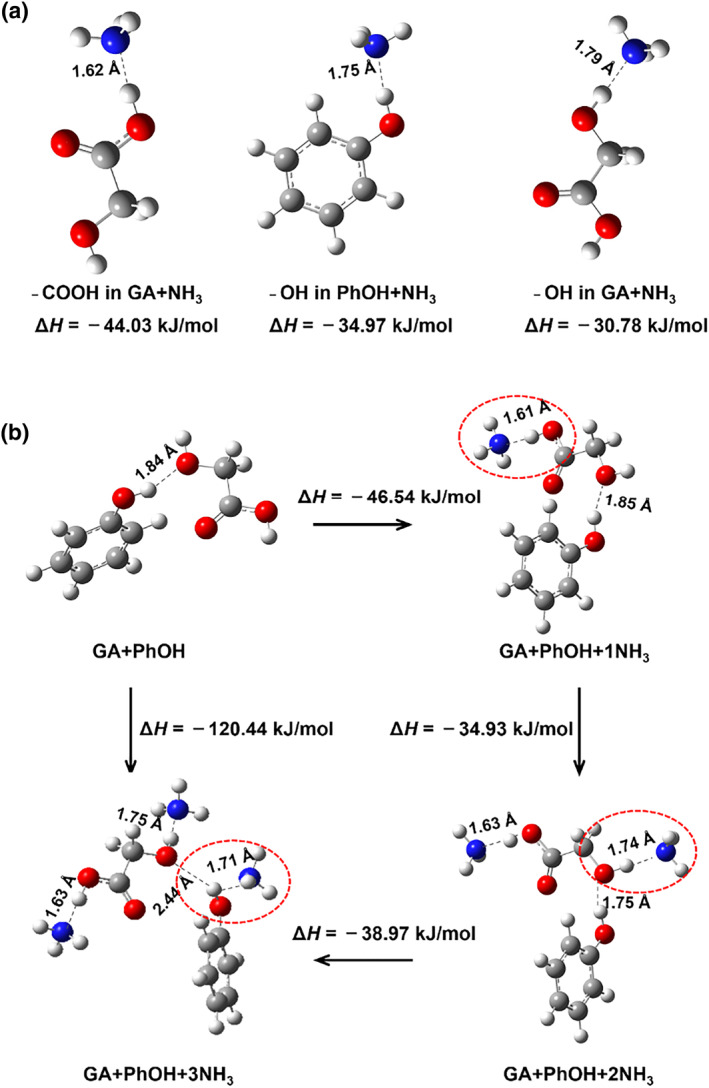
Optimized structures for (a) PhOH + NH_3_ and GA + NH_3_ systems, as well as (b) GA + PhOH and GA + PhOH + NH_3_ systems together with corresponding interaction enthalpies and atom–atom distances (gray: C; red: O; blue: N; white: H). GA, glycolic acid.

Next, the QC and MD theoretical calculations revealed the contribution of GA and PhOH to the high absorption capacity and selective recognition of NH_3_ at the molecular level. The enthalpy changes of the interaction of the three sites −OH in PhOH, −COOH and −OH in GA with NH_3_ were calculated, respectively. As shown in Figure 8a, −OH in PhOH, −COOH and −OH in GA can all interact strongly with NH_3_, which is responsible for the fact that GA + PhOH DESs can recognize NH_3_ efficiently. The enthalpy changes are −34.95 kJ/mol, −46.51 kJ/mol as well as −30.77 kJ/mol, respectively. The interaction of NH_3_ with −COOH in GA is stronger than that with −OH in PhOH since the acidity of PhOH is weaker than that of GA. In addition, there are two types of active sites in GA, which account for the fact that a higher content of GA leads to a higher NH_3_ absorption capacity.

Further, the interaction modes between GA + PhOH DES and NH_3_ in the presence of three types of active sites were evaluated. The structures of the optimized GA + PhOH and GA + PhOH + NH_3_ systems are illustrated in Figure 8b. For the GA + PhOH system, the O atom of −COOH in GA and the H atom of −OH in PhOH form intermolecular hydrogen bonds, which also indicates that PhOH acts as an HBD, and GA acts as a hydrogen bond acceptor (HBA) to form the GA + PhOH DES. For the GA + PhOH + NH_3_ system, in the first step, NH_3_ prefers to attach to −COOH in GA, and the distance of H (−COOH)—N (NH_3_) is 1.61 Å. NH_3_ then binds to −OH in GA in the second step, and the atom‐atom distance is 1.74 Å. In the third step, the intermolecular hydrogen bond between −OH in GA and −OH in PhOH was destroyed, and NH_3_ binds to −OH in PhOH with an atom‐atom distance of 1.71 Å. The enthalpy changes for the three steps are −46.54, −34.93 and −38.97 kJ/mol, respectively, which demonstrates the thermodynamically favorable nature of the three steps. The interaction between −COOH in GA and NH_3_ is an acid‐base interaction, which is also corroborated with the FTIR results. Whereas, the interactions between −OH in PhOH and NH_3_, as well as, −OH in GA and NH_3_ are hydrogen bonding interactions. In addition, these results also indicate the ordering of the strength of the interaction of the three types of active sites with NH_3_ as −COOH in GA > −OH in PhOH > −OH in GA.

Possible modes for the GA + PhOH + CO_2_, GA + PhOH + N_2_ and GA + PhOH + H_2_ systems are also provided in Figures S6–S8. The enthalpy changes and the corresponding atom‐atom distances of the optimal geometries of GA + PhOH + CO_2_, GA + PhOH + N_2_, and GA + PhOH + H_2_ are −4.39 kJ/mol (3.29 Å), −2.78 kJ/mol (3.09 Å), and −2.60 kJ/mol (2.52 Å), respectively. The extremely low enthalpy changes and remarkably long atom‐atom distances indicate that there are no strong interactions between GA + PhOH and CO_2_, N_2_ or H_2_. Thus, the GA + PhOH system is barely able to attract H_2_, N_2_ and CO_2_molecules, accounting for the highly efficient recognition of NH_3_ in NH_3_/CO_2_/N_2_ and NH_3_/N_2_/H_2_ mixtures by GA + PhOH DES.

**FIGURE 9:**
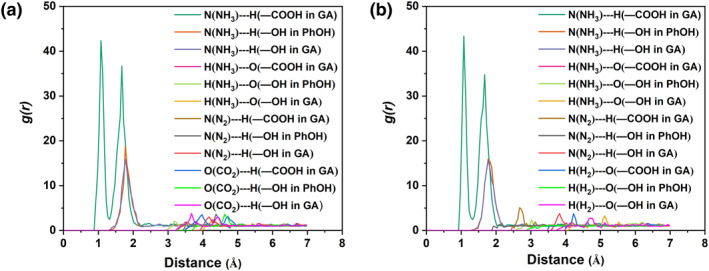
Atom–atom RDFs of the (a) GA + PhOH + NH_3_ + CO_2_ + N_2_ system and (b) GA + PhOH + NH_3_ + N_2_ + H_2_ system. GA, glycolic acid; RDFs, radial distribution functions.

GA + PhOH DESs can interact more strongly with NH_3_ than with H_2_, CO_2_ as well as N_2_, which can also be revealed by atom‐atom RDFs, as shown in Figure 9. For the GA + PhOH + NH_3_ + CO_2_ + N_2_ system, there are two strong signals at 1.075 Å and 1.625 Å for N(NH_3_)‐‐‐H(−COOH in GA). Note that the RDF signals for both N(NH_3_)‐‐‐H(−OH in PhOH) and N(NH_3_)‐‐‐H(−COOH in GA) are located at 1.725 Å, suggesting that NH_3_ interacts more strongly with −COOH in GA than with −OH in PhOH or GA. In addition, the signal of N (NH_3_)‐‐‐H (−OH in PhOH) is slightly stronger than that of N (NH_3_)‐‐‐H (−COOH in GA), which indicates that the hydrogen bonding interaction between NH_3_ interacts more strongly with −OH in PhOH than with −OH in GA, and this is in agreement with the results of QC theoretical calculations. Other atom‐to‐atom RDF signals, particularly those associated with CO_2_, N_2_ and H_2_, can be disregarded. Similar conclusions can be drawn from the RDFs for the GA + PhOH + NH_3_ + N_2_ + H_2_ system. In summary, these results demonstrate the extremely weak interactions of GA + PhOH DES with CO_2_, N_2_, and H_2_, as well as, the highly selective recognition for NH_3_, which is induced by the multiple interactions between GA + PhOH DES and NH_3_.

## 4 CONCLUSIONS

To conclude, a class of GA + PhOH DESs with multiple active sites and low viscosity were fabricated. Their basic physicochemical properties, NH_3_ absorption and selective recognition ability were determined in detail, and the intrinsic mechanism by which GA + PhOH DES interact with NH_3_ was elucidated. The rates of NH_3_ absorption in GA + PhOH DESs are notably rapid, and the NH_3_ absorption behavior is non‐ideal since GA + PhOH DESs can chemically interact with NH_3_ and GA is a more critical component for trapping NH_3_. The solubility of NH_3_ exceeds that of the majority of ILs and DESs. The NH_3_ solubility at low pressure are negligibly affected after 100 days of exposure to air. During 10 after undergoing 10 cycles, the NH_3_ solubility of GA + PhOH DESs remain highly stable. The GA + PhOH DESs display a high selectivity for NH_3_ in mixtures of H_2_, CO_2_ and N_2_. Theoretical calculations confirm the multiple interactions of GA + PhOH DESs with NH_3_, which is responsible for the fact that NH_3_ in gas mixtures can be efficiently recognized and selectively captured by GA + PhOH DESs. On this basis, GA + PhOH DESs are expected to be potential materials for highly selective capture of NH_3_ in synthetic NH_3_ processes and in industrial exhaust streams.

## CONFLICT OF INTEREST STATEMENT

The authors declare no conflicts of interest.

## ETHICS STATEMENT

None.

## Supporting information

Supporting Information S1

## Data Availability

Data will be made available on request.
