# Supplementary material for: Rational design of deep eutectic solvents with low viscosities and multiple active sites for efficient recognition and selective capture of NH3
Source: Smart Mol. 2025 Jan 5;3(1):e20240045. doi: 10.1002/smo.20240045 (PMC12117893; doi:10.1002/smo.20240045)
Supplement: Supplementary file 1 — Supporting Information S1 [file SMO2-3-e20240045-s001.docx]

**SUPPLEMENTARY MATERIAL**

**Rational design of deep eutectic solvents with low viscosities and multiple active sites for efficient recognition and selective capture of NH_3_**

Lu Zheng^a^, Saisai Ju^a^, Siqi Fang^a^, Hongwei Zhang^a^, Zhenping Cai^a^, Kuan Huang^a,b^* and Lilong Jiang^a,b^*

^a^National Engineering Research Center of Chemical Fertilizer Catalyst (NERC-CFC), College of Chemical Engineering, Fuzhou University, Fuzhou, Fujian 350002, China.

^b^Qingyuan Innovation Laboratory, Quanzhou, Fujian 362801, China.

*Corresponding authors: [huangk@fzu.edu.cn](mailto:huangk@fzu.edu.cn) (K. Huang); [jll@fzu.edu.cn](mailto:jll@fzu.edu.cn) (L. Jiang).

Table S1. Moisture contents of GA+PhOH DESs synthesized in this work.

| DES | Moisture content (wt.%) |
| --- | --- |
| GA+PhOH (1:1) | 0.76 |
| GA+PhOH (1:2) | 0.30 |
| GA+PhOH (1:3) | 0.50 |
| GA+PhOH (1:5) | 0.72 |

Table S2. Densities of GA+PhOH DESs at different temperatures.

| Temperature (K) | Density (g/cm^3^) | | | | |
| --- | --- | --- | --- | --- | --- |
|  | GA+PhOH (1:1) | GA+PhOH (1:2) | GA+PhOH (1:3) | GA+PhOH (1:5) | GA+PhOH (1:3)+NH_3_ adsorption |
| 298.2 | 1.1862 | 1.1425 | 1.1226 | 1.1034 | 1.1426 |
| 303.2 | 1.1817 | 1.1382 | 1.1182 | 1.0991 | 1.1384 |
| 308.2 | 1.1773 | 1.1338 | 1.1139 | 1.0948 | 1.1342 |
| 313.2 | 1.1752 | 1.1294 | 1.1095 | 1.0907 | 1.1299 |
| 318.2 | 1.1704 | 1.1249 | 1.1051 | 1.0863 | 1.1257 |
| 323.2 | 1.1646 | 1.1205 | 1.1007 | 1.0816 | 1.1214 |
| 328.2 | 1.1596 | 1.1161 | 1.0963 | 1.0767 | 1.1171 |
| 333.2 | 1.1548 | 1.1116 | 1.0919 | 1.0723 | 1.1128 |
| 338.2 | 1.1499 | 1.1071 | 1.0874 | 1.0679 | 1.1085 |
| 343.2 | 1.1449 | 1.1026 | 1.0829 | 1.0627 | 1.1042 |
| 348.2 | 1.1399 | 1.0981 | 1.0783 | 1.0582 | 1.0999 |
| 353.2 | 1.1342 | 1.0935 | 1.0738 | 1.0537 | 1.0955 |

Table S3. Viscosities of GA+PhOH DESs at different temperatures.

| Temperature (K) | Viscosity (mPa∙s) | | | | |
| --- | --- | --- | --- | --- | --- |
|  | GA+PhOH (1:1) | GA+PhOH (1:2) | GA+PhOH (1:3) | GA+PhOH (1:5) | GA+PhOH (1:3)+NH_3_ absorption |
| 298.2 | 27.95 | 18.01 | 15.17 | 11.27 | 36.32 |
| 303.2 | 20.80 | 13.90 | 11.80 | 9.05 | 26.89 |
| 308.2 | 16.03 | 10.90 | 9.41 | 7.36 | 20.94 |
| 313.2 | 13.37 | 8.71 | 7.67 | 6.11 | 17.93 |
| 318.2 | 10.69 | 7.12 | 6.34 | 5.12 | 15.58 |
| 323.2 | 8.71 | 5.88 | 5.32 | 4.35 | 12.93 |
| 328.2 | 7.20 | 4.95 | 4.53 | 3.70 | 9.74 |
| 333.2 | 6.00 | 4.21 | 3.92 | 3.20 | 8.31 |
| 338.2 | 5.06 | 3.60 | 3.40 | 2.81 | 6.88 |
| 343.2 | 4.31 | 3.13 | 2.98 | 2.47 | 5.86 |
| 348.2 | 3.79 | 2.75 | 2.37 | 2.20 | 5.10 |
| 353.2 | 3.32 | 2.43 | 2.11 | 1.97 | 4.60 |

Table S4. Fitting parameters of Equations 2 and 3.

| Parameter | GA+PhOH (1:1) | GA+PhOH (1:2) | GA+PhOH (1:3) | GA+PhOH (1:5) | GA+PhOH (1:3)+NH_3_ absorption |
| --- | --- | --- | --- | --- | --- |
| *a* (g/cm^3^) | 1.470 | 1.408 | 1.387 | 1.375 | 1.398 |
| *b*×10^-4^ (g/cm^3^·K) | -9.464 | -8.910 | -8.863 | -9.094 | -8.564 |
| *R*^2^ | 0.996 | 0.9999 | 0.9999 | 0.9999 | 0.9999 |
| *η*_0_×10^-3^ (mPa·s) | 4.927 | 4.535 | 3.588 | 3.687 | 3.412 |
| *D* (K) | 697.6 | 651.6 | 719.4 | 714.4 | 927.6 |
| *T*_0_ (K) | 188.0 | 189.3 | 179.1 | 173.7 | 164.7 |
| *R*^2^ | 0.9993 | 0.99999 | 0.9996 | 0.99999 | 0.9943 |

Table S5. Solubilities of NH_3_ in GA+PhOH DESs with different molar ratios of GA to PhOH at 313.2 K.

| GA+PhOH (1:1) | | GA+PhOH (1:2) | | GA+PhOH (1:3) | | GA+PhOH (1:5) | |
| --- | --- | --- | --- | --- | --- | --- | --- |
| Pressure (kPa) | NH_3_ solubility (mol/kg) | Pressure (kPa) | NH_3_ solubility (mol/kg) | Pressure (kPa) | NH_3_ solubility (mol/kg) | Pressure (kPa) | NH_3_ solubility (mol/kg) |
| 0 | 0 | 0 | 0 | 0 | 0 | 0 | 0 |
| 1.5 | 2.05 | 1.5 | 1.80 | 1.6 | 1.66 | 1.3 | 1.58 |
| 2.9 | 3.55 | 3.0 | 3.13 | 2.4 | 2.50 | 3.0 | 2.45 |
| 5.1 | 4.48 | 5.0 | 3.87 | 4.7 | 3.42 | 4.9 | 3.22 |
| 10.7 | 6.75 | 7.5 | 4.59 | 6.7 | 4.18 | 7.9 | 4.10 |
| 20.0 | 8.06 | 9.8 | 5.05 | 8.0 | 4.33 | 10.1 | 4.44 |
| 34.8 | 9.26 | 14.7 | 6.08 | 9.8 | 4.73 | 15.0 | 5.22 |
| 41.2 | 9.64 | 20.1 | 6.87 | 12.9 | 5.30 | 19.8 | 5.90 |
| 61.5 | 10.54 | 32.0 | 7.87 | 15.7 | 5.85 | 25.0 | 6.44 |
| 81.5 | 11.31 | 40.4 | 8.37 | 20.3 | 6.46 | 35.3 | 7.22 |
| 101.4 | 11.97 | 60.0 | 9.29 | 25.3 | 6.94 | 44.9 | 7.80 |
| 128.7 | 12.82 | 79.9 | 10.02 | 35.7 | 7.69 | 64.6 | 8.69 |
| 161.0 | 13.71 | 101.1 | 10.70 | 44.4 | 8.19 | 84.9 | 9.43 |
| 201.0 | 14.72 | 125.0 | 11.39 | 59.8 | 8.91 | 100.3 | 9.94 |
|  |  | 150.3 | 12.04 | 80.4 | 9.67 | 124.3 | 10.63 |
|  |  | 175.8 | 12.67 | 100.3 | 10.32 | 151.1 | 11.32 |
|  |  | 199.9 | 13.24 | 124.6 | 11.02 | 174.4 | 11.89 |
|  |  |  |  | 150.7 | 11.70 | 201.3 | 12.51 |
|  |  |  |  | 175.1 | 12.31 |  |  |
|  |  |  |  | 200.1 | 12.90 |  |  |

Table S6. Comparison of NH_3_ capacities for different solid adsorbents.

| Absorbent | T (K) | P (kPa) | NH_3_ capacity (mol/kg) | Ref. |
| --- | --- | --- | --- | --- |
| GA+PhOH (1:2) | 298.2 | 9.9 | 6.82 | This work |
|  |  | 111.9 | 12.75 |  |
| GA+PhOH (1:1) | 313.2 | 10.7 | 6.75 | This work |
|  |  | 101.4 | 11.97 |  |
| MPTS-1.5 | 298.0 | 10.0 | 4.23 | ^[58]^ |
| Ni_2_Cl_2_BTDD | 298.0 | 10.0 | 5.96 | ^[59]^ |
| Ni_acrylate | 298.0 | 10.0 | 4.76 | ^[59]^ |
| [Bmim]_2_[Co(SCN)_4_]@silica | 303.0 | 10.0 | 3.22 | ^[60]^ |
| SPF−Na | 353.2 | 10.0 | 6.23 | ^[61]^ |
| MFI | 263.0 | 20.0 | 1.34 | ^[62]^ |
| NU-300 | 298 K | 101.1 | 8.28 | ^[63]^ |
| Co_4_ (IDC)_4_(pda)_4_ | 298 K | 101.1 | 11.50 | ^[64]^ |
| MFM-303(Al) | 273 K | 101.1 | 9.90 | ^[65]^ |
| 1TCS | 298 K | 101.1 | 8.52 | ^[66]^ |
| Ni_acryl_TMA | 298 K | 101.1 | 23.5 | ^[59]^ |
| [Fe_2_(H_2_opba)_2_Cl_2_(dmso)_2_]·2CH_2_Cl_2_·2dmso | 298 K | 101.1 | 11.30 | ^[67]^ |
| SION105-Eu | 303 K | 101.1 | 5.70 | ^[68]^ |
| Ga-PMOF | 298 K | 101.1 | 10.50 | ^[69]^ |

Table S7. Solubilities of NH_3_ in GA+PhOH (1:2) at different temperatures.

| 298.2 K | | 313.2 K | | 333.2 K | |
| --- | --- | --- | --- | --- | --- |
| Pressure (kPa) | NH_3_ solubility (mol/kg) | Pressure (kPa) | NH_3_ solubility (mol/kg) | Pressure (kPa) | NH_3_ solubility (mol/kg) |
| 0 | 0 | 0 | 0 | 0 | 0 |
| 1.1 | 3.20 | 1.5 | 1.80 | 2.2 | 2.87 |
| 3.6 | 4.59 | 3.0 | 3.13 | 3.8 | 3.24 |
| 4.7 | 5.19 | 5.0 | 3.87 | 6.0 | 3.73 |
| 6.7 | 5.99 | 7.5 | 4.59 | 9.0 | 4.29 |
| 9.4 | 6.61 | 9.8 | 5.05 | 10.6 | 4.52 |
| 9.9 | 6.82 | 14.7 | 6.08 | 16.2 | 5.23 |
| 16.4 | 7.71 | 20.1 | 6.87 | 25.1 | 5.93 |
| 19.9 | 8.15 | 32.0 | 7.87 | 33.1 | 6.43 |
| 30.5 | 8.98 | 40.4 | 8.37 | 40.7 | 6.84 |
| 41.8 | 9.65 | 60.0 | 9.29 | 62.9 | 7.67 |
| 60.1 | 10.64 | 79.9 | 10.02 | 83.6 | 8.25 |
| 81.2 | 11.61 | 101.1 | 10.70 | 108.3 | 8.85 |
| 111.9 | 12.75 |  |  |  |  |

Table S8. Solubilities of CO_2_ in GA+PhOH (1:2) at different temperatures.

| 298.2 K | | 313.2 K | | 333.2 K | |
| --- | --- | --- | --- | --- | --- |
| Pressure (kPa) | CO_2_ solubility (mol/kg) | Pressure (kPa) | CO_2_ solubility (mol/kg) | Pressure (kPa) | CO_2_ solubility (mol/kg) |
| 0 | 0 | 0 | 0 | 0 | 0 |
| 25.9 | 0.016 | 25.4 | 0.009 | 24.9 | 0.007 |
| 50.5 | 0.026 | 49.7 | 0.021 | 52.8 | 0.018 |
| 76.2 | 0.044 | 75.4 | 0.028 | 76.1 | 0.020 |
| 104 | 0.057 | 100.1 | 0.042 | 103 | 0.029 |
| 150 | 0.079 | 127.2 | 0.056 | 124.1 | 0.034 |
| 175.2 | 0.090 | 149 | 0.063 | 151.2 | 0.043 |
| 213 | 0.104 | 175.5 | 0.072 | 209.8 | 0.061 |
| 249.7 | 0.123 | 200.7 | 0.084 | 249.6 | 0.073 |
|  |  | 250.9 | 0.102 |  |  |

Table S9. NH_3_/CO_2_ selectivities of different ILs and DESs.

| Absorbent | T (K) | P (kPa) | NH_3_/CO_2_ selectivity | Ref. |
| --- | --- | --- | --- | --- |
| GA+PhOH (1:2) | 313.2 | 101.1 | 254.8 | This work |
| [APH]NO_3_-Res (2:1) | 293.0 | 100.0 | 23.6 | ^[37]^ |
| EaCl-Gly(1:2)-6% OHP[5] | 298.2 | 100.0 | 84.0 | ^[41]^ |
| LiCl/EG (1:3) | 303.2 | 101.3 | 389.7 | ^[42]^ |
| GI-AT (1:2) | 303.2 | 100.0 | 151.0 | ^[43]^ |
| MgCl_2_/Res/EG (0.25:1:2) | 303.2 | 101.3 | 76.2 | ^[44]^ |
| [Bmim]_2_[CuCl_4_] | 303.2 | 100 | 50.0 | ^[45]^ |
| [Me_2_COH_2_N]Cl:U (1:1) | 313.2 | 100.0 | 30.0 | ^[49]^ |
| ChCl/Res/Gly (1:3:5) | 313.2 | 101.3 | 369.0 | ^[50]^ |
| [EtOHmim][BF_4_] | 313.2 | 115.6 | 61.4 | ^[14]^ |
| 2-NH_2_Py/Res (1:2) | 313.2 | 101.3 | 92.2 | ^[51]^ |
| TetrZ + 1,2,3- tri(1:4) | 313.2 | 101.3 | 592.7 | ^[52]^ |
| Res/PhOH (1:3) | 303.2 | 101.3 | 93.3 | ^[53]^ |
| [2-mPy][NTf2] | 313.2 | 100.0 | 51.0 | ^[54]^ |
| EaCl/Res/Gly (1:4:5) | 313.2 | 101.3 | 171.8 | ^[55]^ |
| [EtOHim][NTf_2_] | 313.0 | 100.0 | 65.0 | ^[56]^ |
| [1, 2, 3-TrizH_2_][CF_3_SO_3_]_2_ | 313.0 | 100.0 | 142.0 | ^[57]^ |


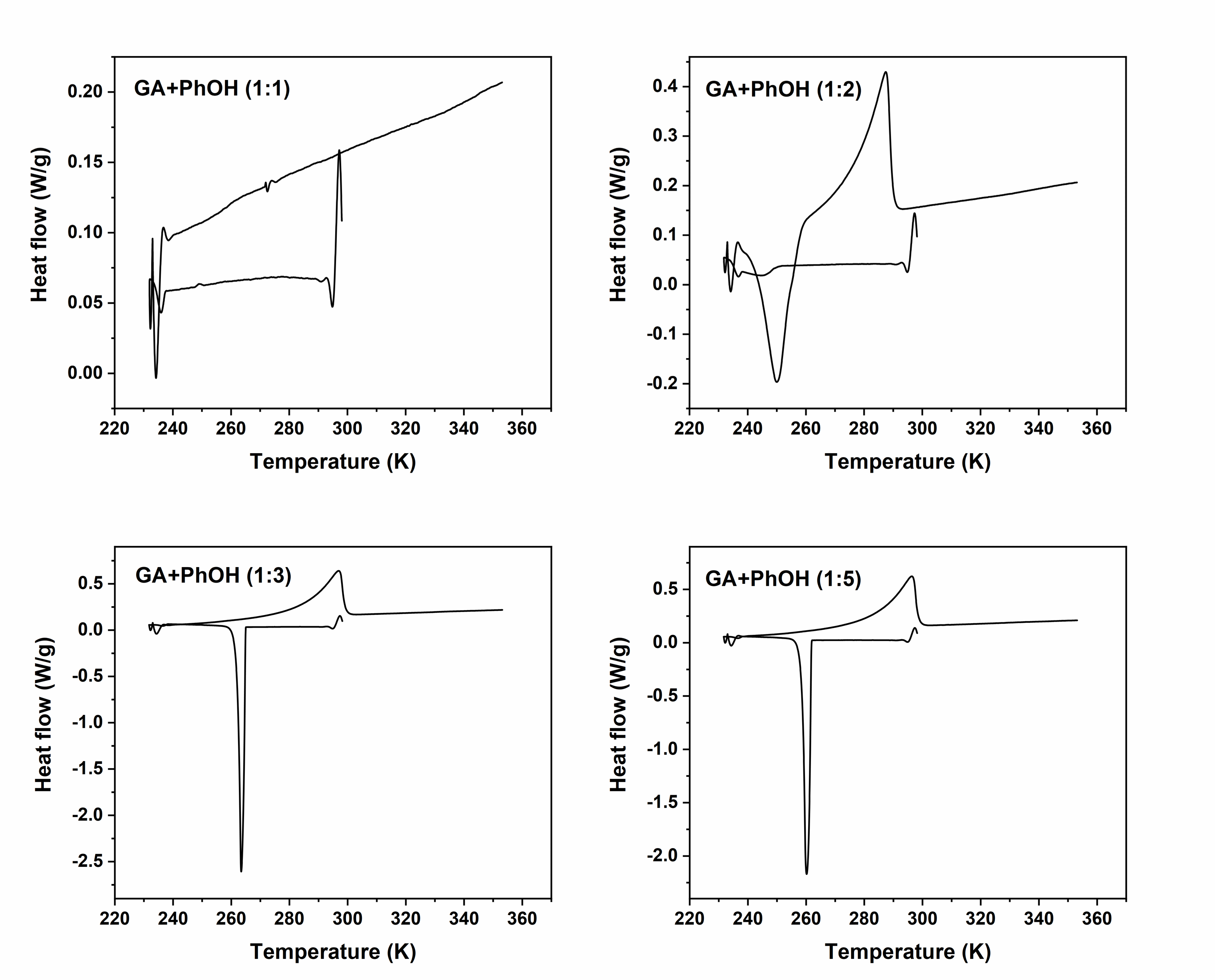


Figure S1. DSC curves of GA+PhOH DESs.





Figure S2. Weight change of GA+PhOH (1:2) under N_2_ purge at 333.2 K.


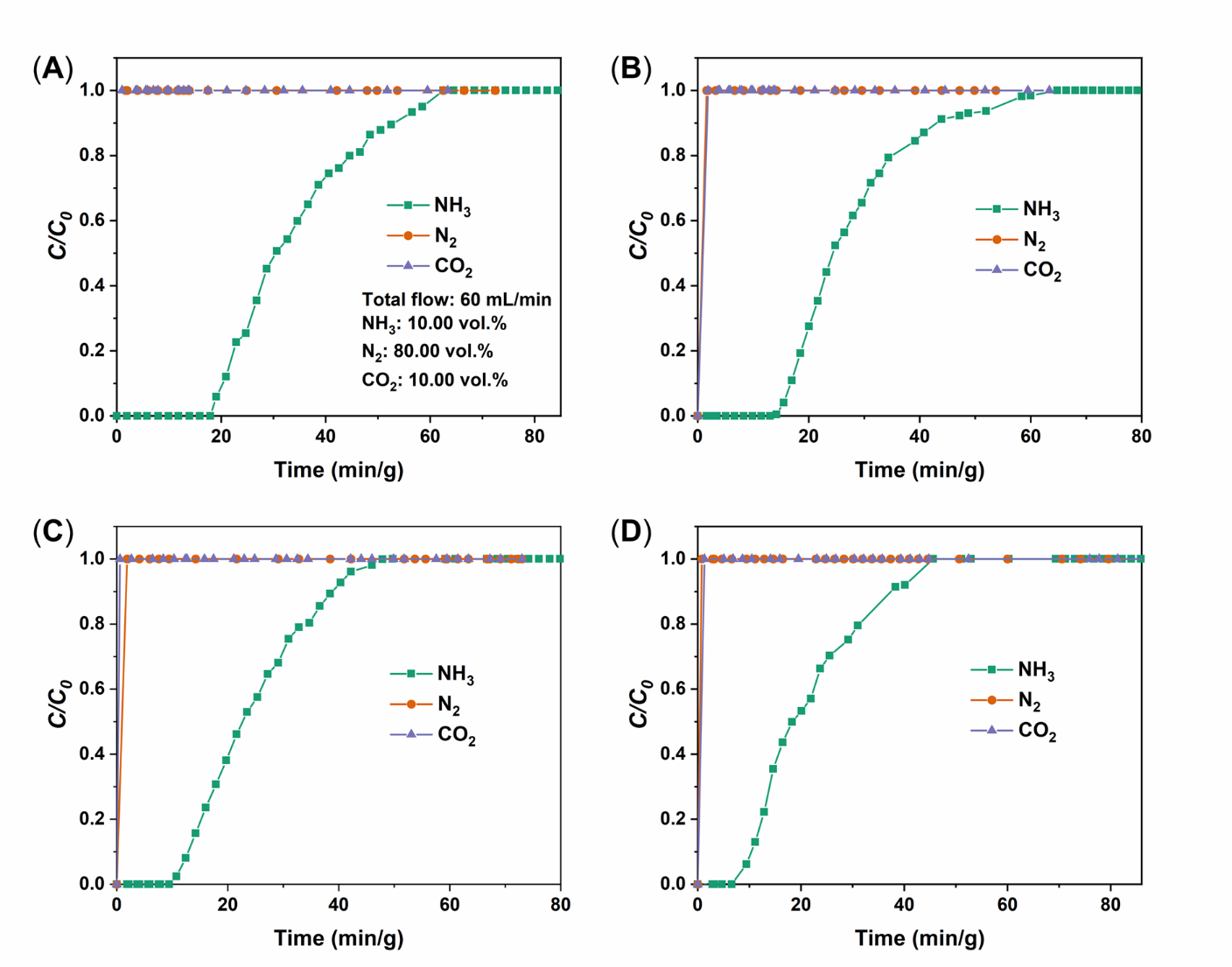


Figure S3. Breakthrough curves for NH_3_/N_2_/CO_2_ mixed gas in (A) GA+PhOH (1:1), (B) GA+PhOH (1:2), (C) GA+PhOH (1:3) and (D) GA+PhOH (1:5) at 313.2 K.





Figure S4. CO_2_ solubilities of GA+PhOH (1:2) at different temperatures.





Figure S5. FTIR spectra of (a) fresh GA+PhOH (1:2), (b) GA+PhOH (1:2) after 14 days of air exposure, (c) fresh GA+PhOH (1:2) after NH_3_ absorption, and (d) GA+PhOH (1:2) after 14 days of exposure to air after NH_3_ absorption.


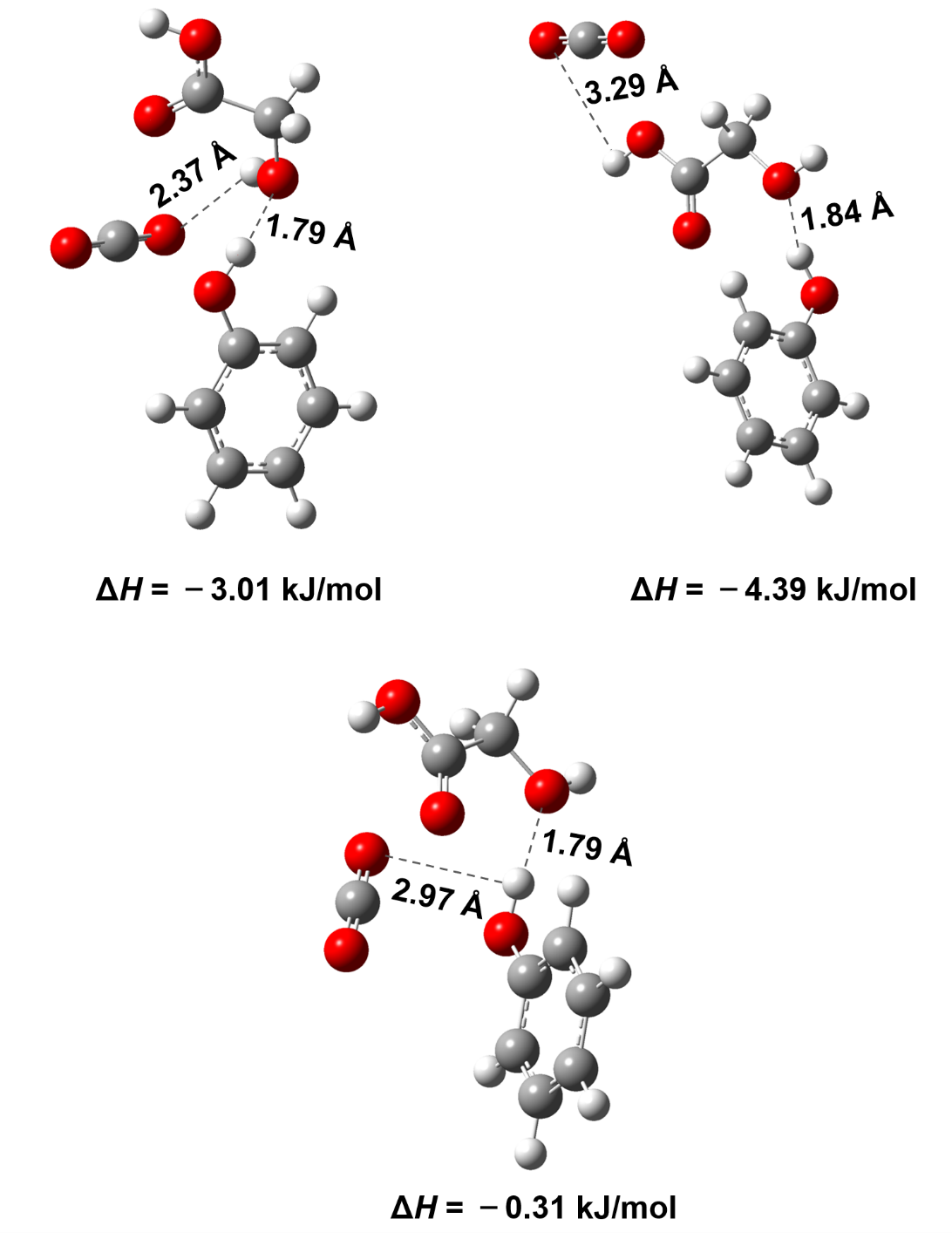


Figure S6. Optimized structures for GA+PhOH+CO_2_ systems together with corresponding interaction enthalpies and atom–atom distances (grey: C; red: O; white: H).


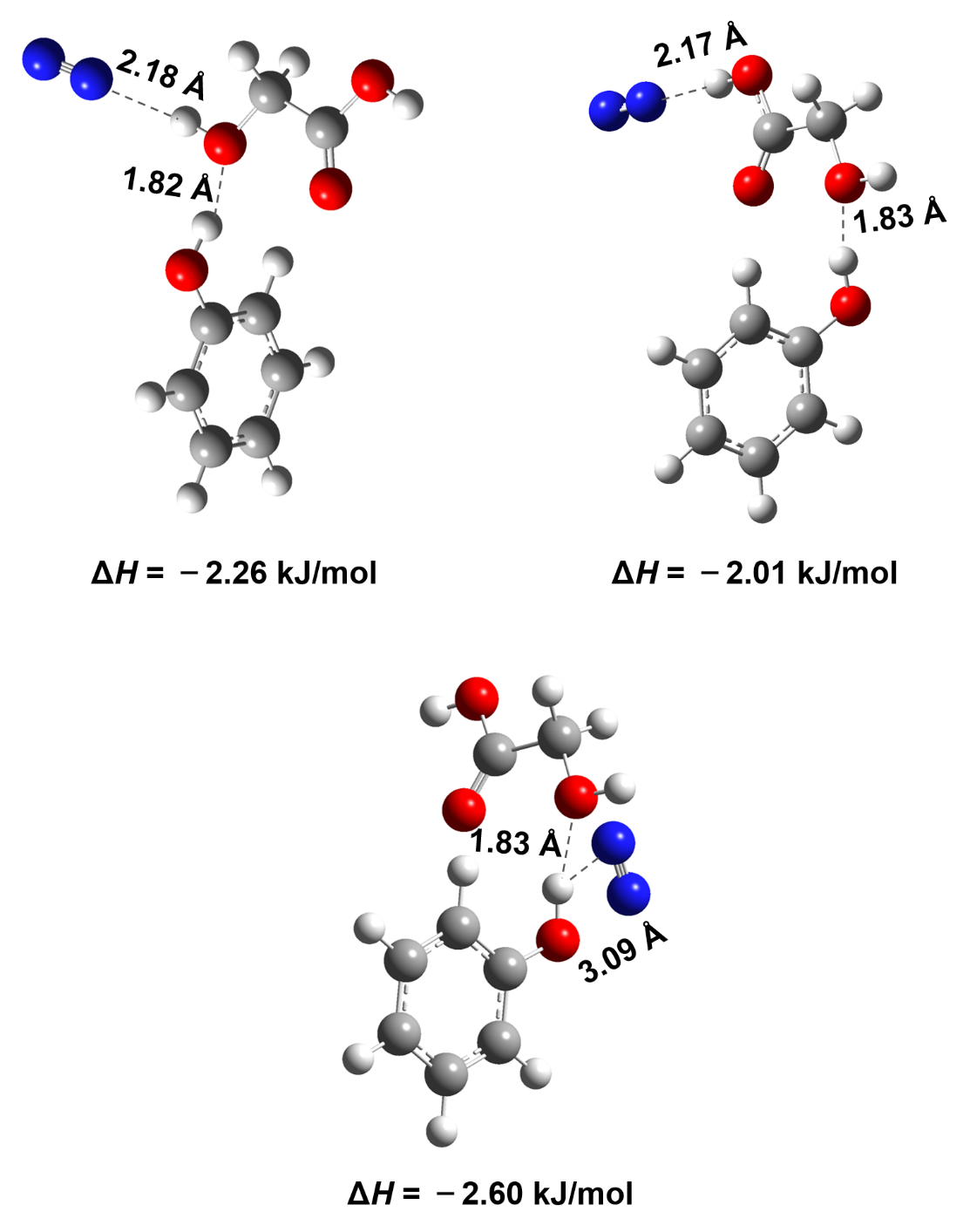


Figure S7. Optimized structures for GA+PhOH+N_2_ systems together with corresponding interaction enthalpies and atom–atom distances (grey: C; red: O; blue: N; white: H).


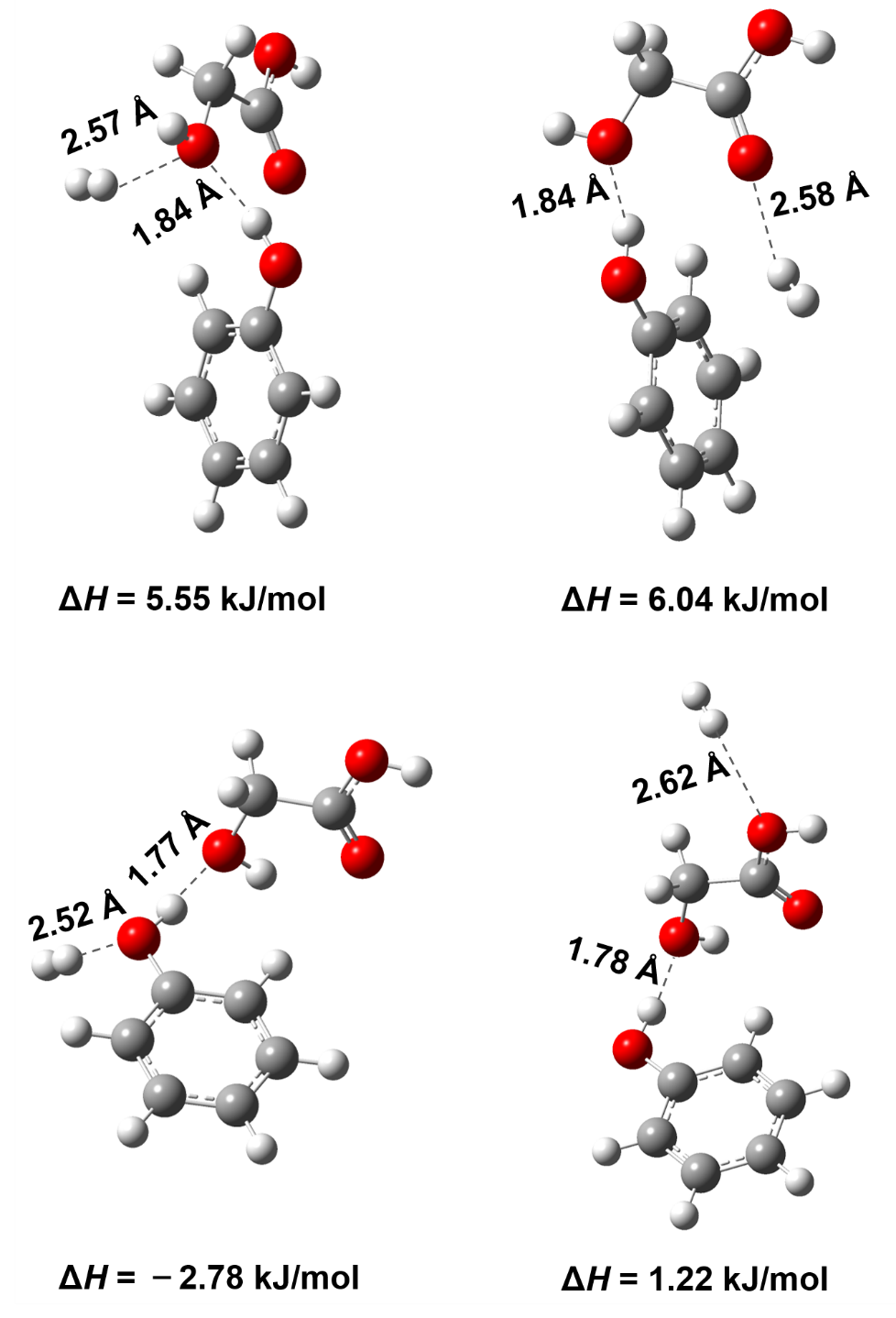


Figure S8. Optimized structures for GA+PhOH+H_2_ systems together with corresponding interaction enthalpies and atom–atom distances (grey: C; red: O; white: H).
